# Dangerous Water-Pipes

**Published:** 1870-12

**Authors:** 


### DANGEROUS WATER-PIPES.

Water-pipes of iron are simple and safe; but with a view to prevent the iron from rusting, and from apparent greater cleanliness, a method has been adopted of lining the interior of the pipe with zinc. In most places this will not only cause the iron to rust sooner, but a chemical decomposition is formed, which causes the formation of some of the most dangerous poisons. Families should everywhere be on their guard against galvanized zinc iron water-pipes, for drinking or cooking purposes.

